# Autoimmune Disease Genetics

**DOI:** 10.1155/2012/262858

**Published:** 2012-12-24

**Authors:** Timothy B. Niewold, George N. Goulielmos, Mohammed Tikly, Shervin Assassi

**Affiliations:** ^1^Division of Rheumatology and Department of Immunology, Mayo Clinic, 200 1st Street SW, Guggenheim Building 3-42, Rochester, MN 55905, USA; ^2^Laboratory of Molecular Medicine and Human Genetics, Department of Medicine, University of Crete, Heraklion, Greece; ^3^Division of Rheumatology, Chris Hani Baragwanath Academic Hospital, University of the Witwatersrand, Johannesburg, South Africa; ^4^Department of Rheumatology, Health Science Center at Houston, University of Texas, Houston, TX 77030, USA

Genetic risk factors play an important role in autoimmune disease susceptibility. Recent advances genotyping techniques, statistical methods, and the organization of large patient cohorts have facilitated explosive progress in this field, and our understanding of the genetic architecture of human autoimmunity is rapidly expanding. Current studies have demonstrated that some genetic risk factors for autoimmunity are shared between diseases [1, 2], and that others may be specific to a particular disease or ancestral background [3, 4]. Knowledge of the genetic basis of disease provides us with a unique window into human pathogenesis, which will facilitate improved diagnostic and therapeutic strategies and enable personalized medicine. It is clear that this field represents a major frontier in human disease research which we are just beginning to understand.

Given this background, we have assembled this special issue with a goal of highlighting important progress in a diverse range of topics in human autoimmune disease. The study designs and topics represented include everything from traditional family-based heritability and candidate analyses which are still clearly relevant and important today to eQTL methods and considerations of how genetic polymorphisms may impact the human immune system. A paper by J. L. Schmidt et al. uses a family-based design to examine the occurrence of autoimmune disease in families with an index case of Aicardi-Goutières Syndrome in the paper entitled “*Family history of autoimmune disease in patients with Aicardi-Goutières Syndrome.*” T. Carvalheiro et al. in the paper “*Tolerogenic versus inflammatory activity of peripheral blood monocytes and dendritic cells subpopulations in systemic lupus erythematosus*” explore the human immune system and find a decrease in tolerogenic dendritic cells in systemic lupus erythematosus, a disease in which dendritic cells play an important role in pathogenesis [5]. Y. Koldobskaya et al. use a novel eQTL technique to prioritize additional candidate gene loci from a prior genome-wide screen of systemic lupus erythematosus, supporting the idea that we can increase the yield of our genetic screens when we can apply our knowledge of disease biology in candidate selection in the paper “*Gene-expression-guided selection of candidate loci and molecular phenotype analyses enhance genetic discovery in systemic lupus erythematosus.*” M. Dolcino et al. profile gene expression in the autoimmune disease dermatitis herpetiformis, demonstrating patterns associated with lesional skin in the paper entitled “*Gene expression profiling in dermatitis herpetiformis skin lesions*.” A candidate gene study by M. Pesmatzoglou et al. provides evidence for genetic associations with immune-mediated thrombocytopenia in the island population of Crete entitled “*DNA methyltransferase 3B gene promoter and interleukin-1 receptor antagonist polymorphisms in childhood immune thrombocytopenia*.” Other studies support the association of a polymorphism in the human type 2 deiodinase gene with disease severity and rate of remission in patients with Grave's disease in the paper “*Thr92Ala polymorphism of human type 2 deiodinase gene (hd2) affects the development of graves' disease, treatment efficiency, and rate of remission*,” and association of polymorphisms in the SPP1 locus with multiple sclerosis in the Italian population in the paper “*The impact of osteopontin gene variations on multiple sclerosis development and progression*.” Z. Zagoriti et al. explore genetic associations with myasthenia gravis in the Hellenic population in the paper entitled “*Genetics of myasthenia gravis: a case-control association study in the hellenic population*.” A. Lev et al. examine the characteristics of residual circulating T cells in the genetic syndrome severe combined immunodeficiency disorder, an immunodeficiency syndrome that can also sometimes demonstrate autoimmune manifestations in the paper “*Characterizing T cells in SCID patients presenting with reactive or residual T lymphocytes*.” A paper by I. A. Sobenin et al. is an example of the diversity of topics covered, as they demonstrate that mitochondrial inheritance influences atherosclerotic disease “*Mitochondrial mutations are associated with atherosclerotic lesions in the human aorta*,” an important condition that clearly has an immune-mediated and inflammatory component. 

Review articles cover diverse topics, such as the role of coinhibitory molecules in autoimmune disease in “*Coinhibitory molecules in autoimmune diseases*,” expression of the autoimmune regulator gene and its impact on immune tolerance in “*Expression of the autoimmune regulator gene and its relevance to the mechanisms of central and peripheral tolerance,*” and genetic factors associated with immune-mediated bone marrow failure syndromes in “*Genetic associations in acquired immune-mediated bone marrow failure syndromes: insights in aplastic anemia and chronic idiopathic neutropenia.*” T. Besenyei et al. review the overlap between human rheumatoid arthritis risk alleles and the corresponding risk loci in animal models of arthritis in the paper “*Non-MHC risk alleles in rheumatoid arthritis and in syntenic chromosome regions of corresponding animal models.*” Two review articles address systemic lupus erythematosus—one is a synthesis of genetic loci associated with the disease that impact monocyte pathways which is “*Genetics of SLE: functional relevance for monocytes/macrophages in disease*,” and the other is a review of the pathogenic influence of the IRF5 locus in systemic lupus erythematosus which is “*Interferon regulatory factor 5 in the pathogenesis of systemic lupus erythematosus*.” While the topics covered in this issue are diverse, they still represent a relatively small portion of the work being done in the large and rapidly moving field of autoimmune disease genetics. It is exciting to observe the ways in which genetic studies are steadily unraveling human autoimmune disease pathogenesis, and the papers presented in this issue contribute to this goal. 

*Timothy B. Niewold* *George N. Goulielmos* *Mohammed Tikly* *Shervin Assassi*
